# Synthesis and evaluation of analogues of the tuberculosis drug bedaquiline containing heterocyclic B-ring units

**DOI:** 10.1016/j.bmcl.2017.10.042

**Published:** 2017-12-01

**Authors:** Peter J. Choi, Hamish S. Sutherland, Amy S.T. Tong, Adrian Blaser, Scott G. Franzblau, Christopher B. Cooper, Manisha U. Lotlikar, Anna M. Upton, Jerome Guillemont, Magali Motte, Laurence Queguiner, Koen Andries, Walter Van den Broeck, William A. Denny, Brian D. Palmer

**Affiliations:** a Auckland Cancer Society Research Centre, School of Medical Sciences, University of Auckland, Private Bag 92019, Auckland 1142, New Zealand; b Maurice Wilkins Centre, University of Auckland, Private Bag 92019, Auckland 1142, New Zealand; c Institute for Tuberculosis Research, College of Pharmacy, University of Illinois at Chicago, 833 South Wood Street, Chicago, IL 60612, USA; d Global Alliance for TB Drug Development, 40 Wall St, New York 10005, USA; e Medicinal Chemistry Department (Infectious Diseases), Janssen Pharmaceuticals, Campus de Maigremont, BP315, 27106 Val de Reuil Cedex, France; f Janssen Infectious Diseases BVBA, Beerse, Belgium

**Keywords:** Bedaquiline, Bedaquiline analogues, Tuberculosis, Drug development

## Abstract

Analogues of bedaquiline where the phenyl B-unit was replaced with monocyclic heterocycles of widely differing lipophilicity (thiophenes, furans, pyridines) were synthesised and evaluated. While there was an expected broad positive correlation between lipophilicity and anti-TB activity, the 4-pyridyl derivatives appeared to have an additional contribution to antibacterial potency. The majority of the compounds were (desirably) more polar and had higher rates of clearance than bedaquiline, and showed acceptable oral bioavailability, but there was only limited (and unpredictable) improvement in their hERG liability.

## Graphical abstract

**Figure:**
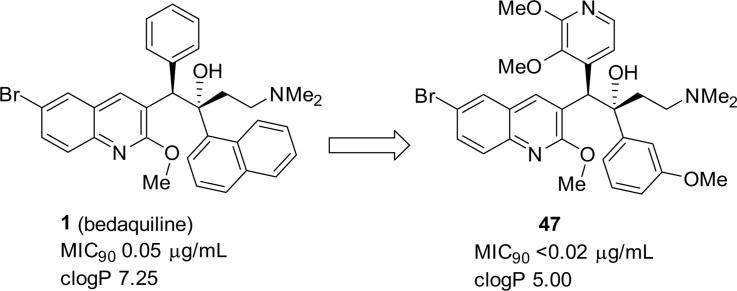

The often late detection of tuberculosis (TB),^1^ coupled with the need to use long and complex multi-drug treatment regimens, has led to an alarming increase in cases that are resistant to the standard front-line drugs (multi-drug-resistant; MDR). In 2016, about 580,000 new cases (3.9% of new cases and 21% of recurrent cases) were classified globally as MDR-TB, and this proportion has been rising rapidly.^2^

Thus the discovery of bedaquiline (TMC207, Sirturo, Janssen Pharmaceuticals; Fig. 1; **1**), a new TB drug, that, due to a novel mechanism of action (inhibition of the mycobacterial ATP synthase^3^) is useful against drug-resistant tuberculosis (MDR-TB), has been of great significance. When added to standard background therapy used for MDR-TB, it demonstrated more rapid bactericidal activity than background therapy alone,^4^ and was approved by the US Food and Drug Administration in 2012 for the treatment of MDR-TB. However it is very lipophilic (measured log P 7.25),^5^ which likely contributes to its long terminal half-life of 5–6 months.^6^ The resultant potential for over-proportional accumulation in tissue has limited the full exploration of its potential dose range.^7^ More generally, highly lipophilic drugs also have a propensity for liver toxicity.^8^ Bedaquiline also shows inhibition of the hERG (human Ether-à-go-go-Related Gene; KCNH2) cardiac potassium channel, with the concomitant risk of delayed ventricular repolarization (QTc interval),^6^ and this is a point to be considered in the planning of combination regimens with other TB drugs with similar effects (e.g. fluoroquinolones, clofazimine).^9^

**Fig. 1:**
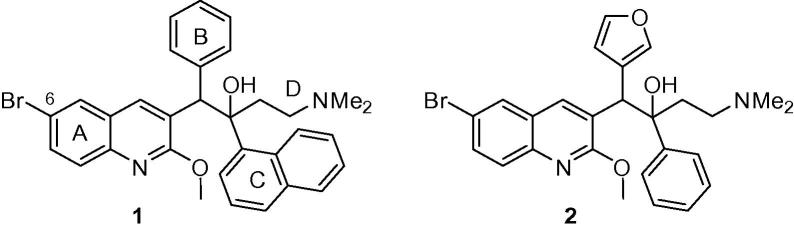
Structure of bedaquiline (**1**) and a furan analogue (**2**).

Thus bedaquiline analogues of comparable antibacterial activity but with lower clog P and less potent inhibition of the hERG potassium channel, but similar in anti-Mtb potency to bedaquiline, would be of substantial interest. We have previously^10^ explored the effects of a range of more polar 6-substituents on the quinoline ring and showed that, on balance, a 6-CN group offered a significant reduction in overall lipophilicity over the standard 6-Br group (about 1.25 log P units) with the least deleterious effect on antibacterial potency.

In the current paper we explore the effects of replacing the phenyl B-ring unit of bedaquiline with heterocycles of differing lipophilicity. One example of such a heterocyclic analogue, the 3-furan **2** also with a smaller (phenyl) C-ring unit, (Fig. 1) has already been reported to show significant activity in *M. smegmatis*, with the separated racemic diastereomer pairs (configurations not assigned) having values of 1.57 and 0.06 µg/mL.^11^

The bedaquiline analogues were synthesized from appropriate benzylquinoline A/B-units and 3-(dimethylamino)-1-arylpropan-1-one C/D-units, following a route described previously.^7^, ^10^ Scheme 1 shows the synthesis of the thienyl A/B-units **54**, **58**, **61** and **64** by base-catalysed condensation (LiTMP) of quinoline **51** and the appropriate thiophene aldehydes^12^, ^13^ **52**,^14^ **56**, **59** and **62** to give the intermediate alcohols **53**, **57**, **60** and **63** in acceptable yields (∼55%). These were then deoxygenated by Et_3_SiH under acid conditions in good yields to the A/B-units **54**, **58**, **61** and **64** (see Supplementary Material for further details).

**Scheme 1:**
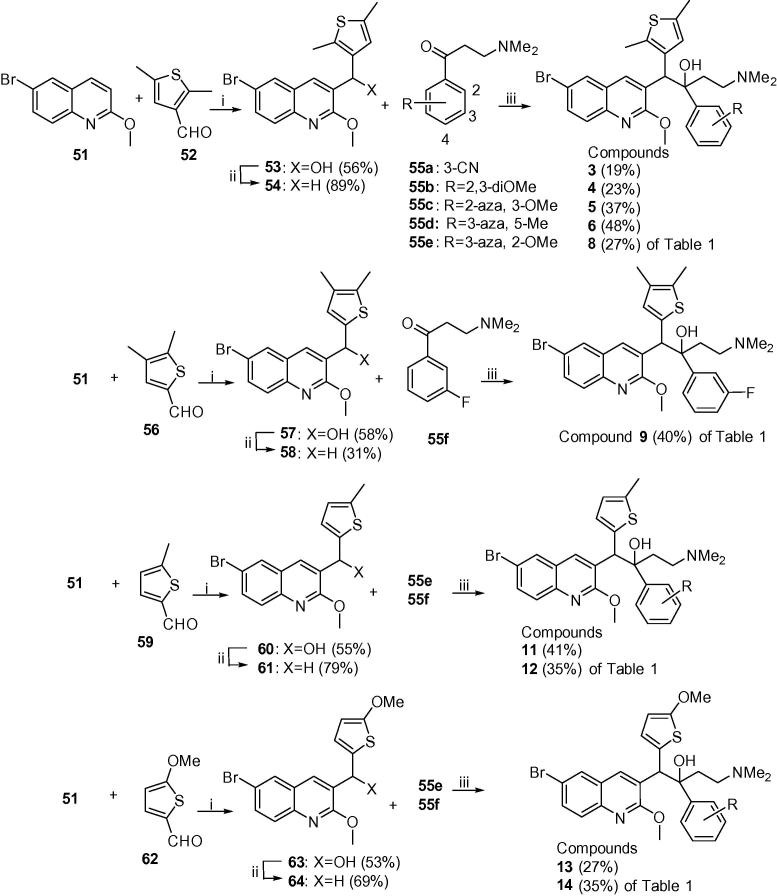
Classes A–D (compounds **3**–**14**). Reagents and conditions: (i) LiTMP, THF, −75 °C, 1.5 h then appropriate aldehyde, −75 °C, 4 h; (ii) Et_3_SiH, TFA, DCM, 20 °C; (iii) LDA or LiTMP, THF, −75 °C, 1.5 h then **55a**–**55f**, −75 °C, 4 h.

In Scheme 2, the 2-furyl analogues **15**–**21** were prepared by the same procedure as in Scheme 1, reacting **51** and 2-furylaldehye (**65**) to give alcohol **66**, which was deoxygenated to give A/B-unit **67**. Condensation of this with the appropriate Mannich bases (selected from **55a**–**55j**) gave compounds **15**–**21**. The 3-furyl analogues **22**–**28** were synthesized by preparation of the boronic acid **68** (from **51**) and Suzuki coupling of this and 3-(bromomethyl)furan **69** to give A/B-unit **70**, and subsequent condensation with the appropriate Mannich base as above (see Supplementary Material for further details).

**Scheme 2:**
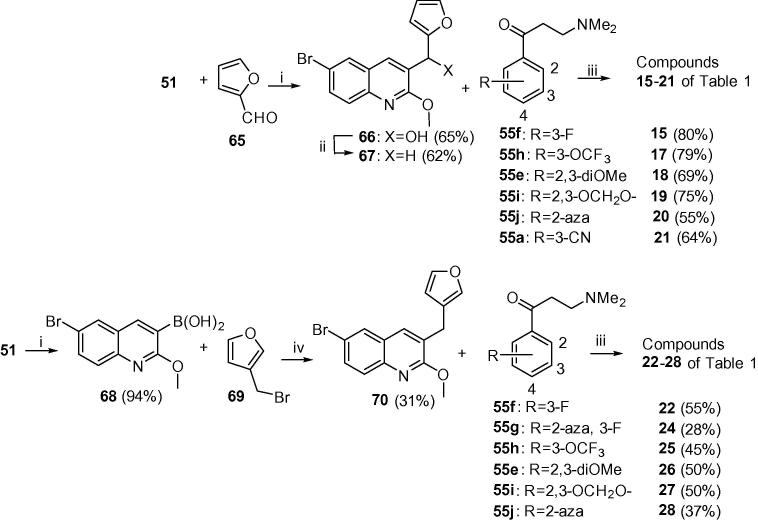
Classes E and F (compounds **15**–**28**). Reagents and conditions: (i) LiTMP, B(OiPr)_3_; (ii) Et_3_SiH, TFA, DCM, 20 °C, 1 h; (iii) LDA, THF, −75 °C, 1.5 h then appropriate ketone **55**, −75 °C, 4 h; (iv) Pd(PPh_3_)_4_, Cs_2_CO_3_, toluene/DMF, 90 °C, 5 h.

Scheme 3, Scheme 4 outline the syntheses of the B-ring unit pyridyl compounds **29**–**50**. In Scheme 3, Suzuki coupling of boronic acid **68** and 2-(chloromethyl)-6-methoxypyridine (**71**) gave A/B-unit **72**, which was condensed with Mannich bases **55a**, **55b** and **55f** and **55l** to give compounds **29**, **30** and **33** of Table 1. Similar reaction of **68** with bromides **82** and **83** gave respectively A/B-units **84** and **85**, which yielded compounds **38** and **39** of Table 1. Compound **35** was prepared by Suzuki coupling of quinoline **51** with aldehyde **73** to give the resulting hydroxyl intermediate **74**. Mesylation followed by reduction of **74** afforded the A/B-unit **75**. Likewise, compounds **36** and **37** were prepared by Suzuki coupling of quinoline **51** with aldehydes **76** and **77** to give the resulting hydroxy intermediates **78** and **79**. Lewis acid mediated deoxygenation of these gave A/B-units **79** and **80** respectively. All of these A/B-units were condensed with Mannich base **55f** to give the target compounds (see Supplementary Material for further details).

**Scheme 3:**
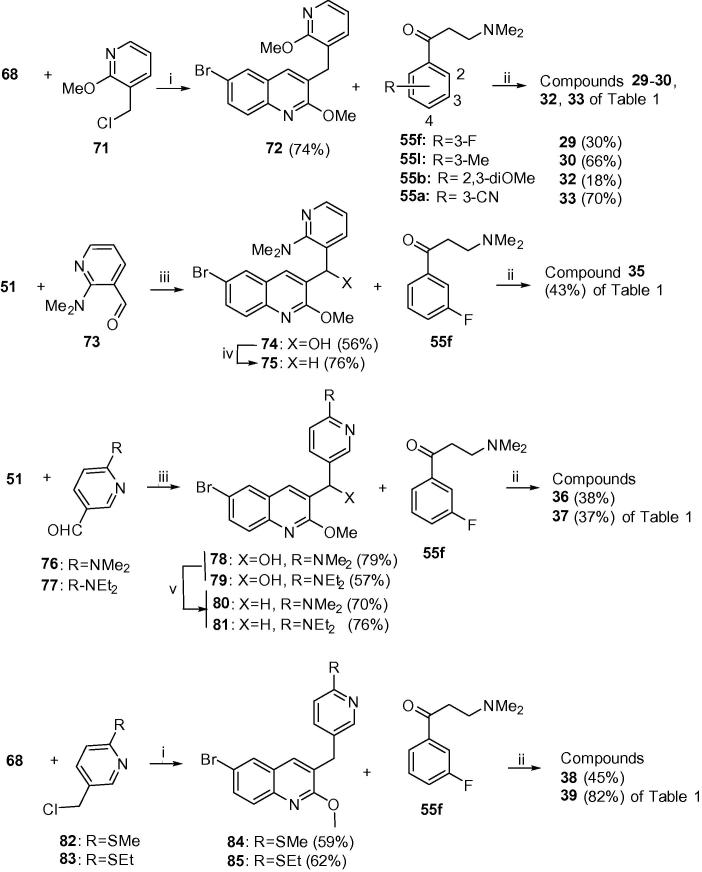
Class G (compounds **29**–**39**). Reagents and conditions: (i) Pd(PPh_3_)_4_, Cs_2_CO_3_, toluene/DMF, reflux; (ii) LDA, THF, −75 °C, 1.5 h then the appropriate ketone **55**, −75 °C, 4 h; (iii) LiTMP, THF, −75 °C, 1.5 h then appropriate aldehyde, −75 °C, 4 h; (iv) MsCl, Et_3_N, DMF, then NaBH_4_; (v) NaBH_4_, AlCl_3_.

**Scheme 4:**
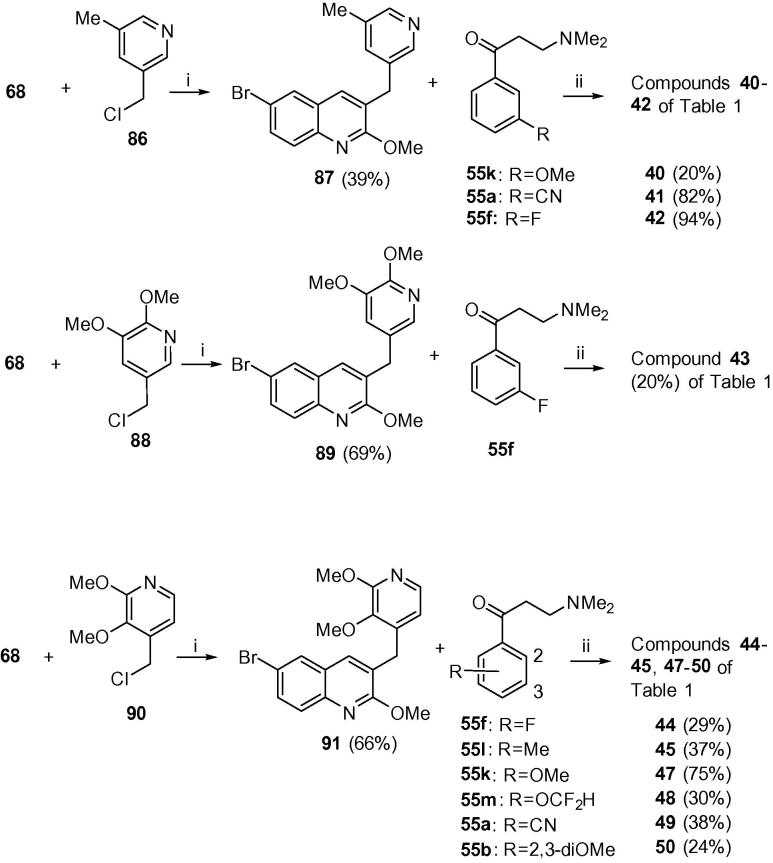
Classes G and H (compounds **40**–**45** and **47**–**50**). Reagents and conditions: (i) Pd(PPh_3_)_4_, Cs_2_CO_3_, toluene/DMF, reflux, 5 h (72%); (ii) LDA, THF, −75 °C, 1.5 h then the appropriate ketone **55**, −75 °C, 4 h.

**Table 1:** Bedaquiline analogues containing heterocyclic ring units.

**Figure:**
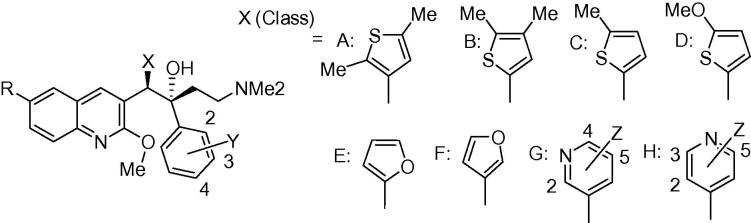

No	R	X (Class)	Y	MIC_90_ (µg/mL)^a^		clog P^b^
				MABA	LORA	
**1**	Br	Phenyl	2,3-Benz	0.05	0.08	7.25
**3**	Br	A	3-CN	0.12	0.12	6.10
**4**	Br	A	2,3-DiOMe	0.50	0.27	6.33
**5**	Br	A	2-Aza, 3-OMe	0.12	0.15	5.99
**6**	Br	A	3-Aza, 5-Me	0.58	0.30	5.67
**7**	CN	A	3-Aza, 5-Me	1.94	1.10	4.24
**8**	Br	A	3-Aza, 2-OMe	0.12	0.20	5.99
**9**	Br	B	3-F	0.06	0.11	6.81
**10**	CN	B	3-F	0.05	0.20	4.67
**11**^c^	Br	C	3-F	0.47^d^		5.01
**12**	Br	C	3-Aza, 2-OMe	0.22	0.52	5.55
**13**	Br	D	3-Aza, 2-OMe	0.24	0.53	5.09
**14**^c^	CN	D	3-F	0.70^d^		4.55
**15**^c^	Br	E	3-F	0.74^d^		5.40
**16**^c^	CN	E	3-F	1.79^d^		4.02
**17**^c^	Br	E	3-OCF_3_	0.76^d^		7.51
**18**^c^	Br	E	2,3-DiOMe	0.71^d^		6.14
**19**^c^	Br	E	2,3-OCH_2_O-	0.69^d^		6.45
**20**^c^	Br	E	2-Aza	1.95^d^		4.98
**21**^c^	Br	E	3-CN	1.96^d^		4.69
**22**^c^	Br	F	3-F	0.74^d^		5.40
**23**^c^	CN	F	3-F	1.08^d^		4.04
**24**^c^	Br	F	2-Aza, 3-F	0.68^d^		5.00
**25**^c^	Br	F	3-OCF_3_	0.76^d^		7.30
**26**^c^	Br	F	2,3-DiOMe	1.16^d^		5.93
**27**^c^	Br	F	2,3-OCH_2_O–	0.90^d^		6.23
**28**^c^	Br	F	2-Aza	1.80^d^		4.77
**29**^c^	Br	G; Z = 2-OMe	3-F	0.12^d^		5.14
**30**	Br	G; Z = 2-OMe	3-Me	0.13	0.25	5.50
**31**	CN	G; Z = 2-OMe	3-Me	0.30	0.15	4.16
**32**^c^	Br	G; Z = 2-OMe	2,3-DiOMe	0.24^d^		4.66
**33**	Br	G; Z = 2-OMe	3-CN	0.34	0.75	4.43
**34**	CN	G; Z = 2-OMe	3-CN	0.38	0.82	3.07
**35**	Br	G; Z = 2-NMe_2_	3-F	0.18	0.20	5.44
**36**	Br	G; Z = 4-NMe_2_	3-F	0.08	0.15	5.44
**37**	Br	G; Z = 4-NEt_2_	3-F	0.02	0.02	6.49
**38**	Br	G; Z = 4-SMe	3-F	0.14	0.37	5.86
**39**	Br	G; Z = 4-SEt	3-F	0.14	0.15	6.39
**40**	Br	G; Z = 5-Me	3-OMe	1.9	2.2	5.00
**41**	Br	G; Z = 5-Me	3-CN	1.32	3.38	4.51
**42**	Br	G; Z = 5-Me	3-F	0.53	0.97	5.22
**43**	Br	G; Z = 4,5-diOMe	3-F	0.47	0.54	5.59
**44**	Br	H; Z = 2,3-diOMe	3-F	0.02	0.07	5.19
**45**	Br	H; Z = 2,3-diOMe	3-Me	0.02	0.04	5.55
**46**	CN	H; Z = 2,3-diOMe	3-Me	0.15	0.14	4.19
**47**	Br	H; Z = 2,3-diOMe	3-OMe	<0.02	<0.02	5.00
**48**	Br	H; Z = 2,3-diOMe	3-OCF_2_H	<0.02	<0.02	5.41
**49**	Br	H; Z = 2,3-diOMe	3-CN	0.13	0.21	4.48
**50**	Br	H; Z = 2,3-diOMe	2,3-DiOMe	0.21^d^		4.71

a MIC_90_ (μg/mL); minimum inhibitory concentration for 90% inhibition of growth of *M.tb* strain H37Rv, determined under aerobic (replicating; MABA) (Ref. 16) or non-replicating (LORA) (Ref. 17) conditions, determined at the Institute for Tuberculosis Research, University of Illinois at Chicago. Each value is the mean of at least two independent determinations.

b clog P calculated by ChemDraw Ultra v12.0.2. (CambridgeSoft).

c RS/SR racemic mixture.

d MICs determined at Tibotec (Mechelen, Belgium).

In Scheme 4, similar Suzuki coupling of boronic acid **68** with chlorides **86**, **88** and **90** gave A/B-units **87**, **89** and **91**. These were condensed with the appropriate Mannich bases to give compounds **40**–**45** and **47**–**50** of Table 1 (see Supplementary Material for further details).

Some of the C/D-unit Mannich bases required have been previously reported.^10^, ^15^ New analogues were prepared either via the Mannich reaction from the corresponding acetophenones or the Grignard reaction from the corresponding Weinreb amides as shown in Scheme 5 (see Supplementary Material for further details).

**Scheme 5:**
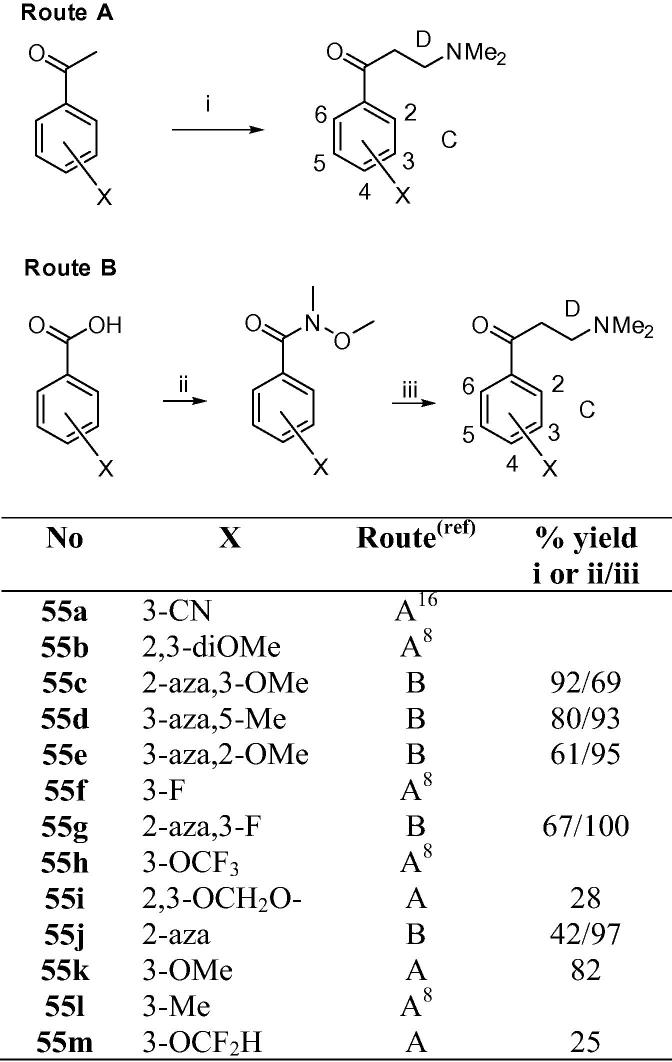
Preparation of C/D-unit Mannich bases. Footnotes for Scheme 5: (i) CH_2_O, Me_2_N·HCl, c·HCl, EtOH, 90 °C, 18 h; (ii) (COCl)_2_, cat. DMF, DCM, then MeNH(OMe)·HCl, pyridine; (iii) vinylMgBr, THF, then Me_2_NH, H_2_O.

For comparison of 6-Br and 6-CN substituents in the A-unit, several Br analogues (**6**, **9**, **15**, **22**, **30**, **33**, **45**) were converted directly to the corresponding CN compounds (**7**, **10**, **16**, **23, 31**, **34**, **46**) (Scheme 6). We have previously shown^10^ that the optimum conditions for this reaction are aryl bromide (1 equiv), zinc (0.1 equiv), zinc (II) cyanide (0.55 equiv), tris(dibenzylideneacetone)dipalladium(0) (Pd_2_(dba)_3_ (0.1 equiv), and tri(*o*-tolyl) phosphine (0.2 equiv).

**Scheme 6:**
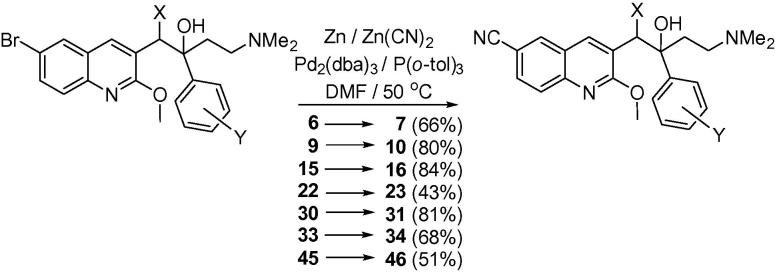
Preparation of cyano analogues.

Reaction of the A/B units prepared in Scheme 1, Scheme 2 with *in situ-*prepared LDA, (lithium diisopropylamide) or LiTMP (lithium tetramethylpiperidide) at −78 °C for 60–90 min) followed by addition of the appropriate Mannich bases **55a**–**j** (−78 °C, 3–4 h, then AcOH) under previously-reported^7^, ^1^ conditions gave required diarylquinolines **3**–**6**, **8**, **9** and **11**–**14** of Table 1, as a racemic mixture of two diastereomers (see Supplementary Material for further details). This mixture was purified by column chromatography to >95% purity in-house, and the desired *RS*, *SR* enantiomer was then separated from the mixture by preparative super-critical fluid HPLC at BioDuro LLC (Beijing). The coupling yields for classes E–H appear to be higher than classes A–D, although this comparison is complicated by the small sample sizes in classes B, C and D. Moreover, in some cases, the coupling reaction proceeded in high conversion, but due to difficulty in purifying the product from impurities the final yield was reduced. There is however, an apparent correlation between the electron density at the benzylic position of the A/B-unit and the yield of the reaction. The best coupling unit (E) is a 2-substituted furan which exerts a strong electron donating effect towards the benzylic position. While the second best unit (G) is a more electron withdrawing pyridine, the 2 or 4-substituted electron donating substituent contributes some electron density towards the benzylic position. Unit H has a 4-aza atom which renders the benzylic position less electron rich, despite a 2-methoxy substituent, and the average coupling yield is even lower. These observations suggest that the lithium anion formed at the benzylic position could be more nucleophilic with a higher electron donation from the B-ring, making the A/B-unit more reactive and favouring the coupling reaction.

The compounds were evaluated for their inhibition of growth (measured as MIC_90_ values in µg/mL) against *M.tb* (strain H37Rv) under both replicating (MABA)^16^ or non-replicating (LORA)^17^ conditions. Under these conditions bedaquiline (**1**) is a potent inhibitor of both (MICs 0.05 and 0.08 µM respectively). In a previous structure-activity relationship (SAR) study of bedaquiline analogues^7^ it was shown that electron-withdrawing groups, especially F or Cl at the 3- and 4-positions on the phenyl B-ring unit (Fig. 1) provided compounds with better MICs against *M. smegmatis*, but at the expense of even higher overall lipophilicity. In a search for less lipophilic analogues, we prepared and evaluated compounds where this phenyl B-ring unit was replaced with different heterocycles of varying lipophilicity (Table 1). A representative subset of compounds were also evaluated for a number of pharmacological properties, and compared against bedaquiline (**1**) (Table 2).

**Table 2:** Comparative activity and pharmacologic data for selected representative analogues.

No	CYP^a^	hERG^b^	Cl_int_^c^	HLM t1/2^d^	F(%)^e^	clog P^f^
**1**	>50	0.37	3	231	70	7.25
**9**	10	0.88	12	60	48	6.81
**35**	>50		45	15		3.08
**36**	>10	0.89	38	18	45	5.44
**39**	10	0.46	11	64	30	6.39
**42**	4.6		56	12	24	5.22
**43**	>10	1.9	36	19	36	5.59
**45**	>10	0.53	13	52	40	5.55
**48**	>10		11	61		4.19
**49**	>10		19	37	68	4.48

a IC_50_ (µM) for inhibition of CYP 3A4 (20 min exposure).

b IC_50_ (µM) for inhibition of the hERG channel; studies conducted by Wuxi AppTec (Ref. 18).

c Cl_int_ (µL/min/mg/protein) in human liver microsomes after 60 min exposure to 1 mg/mL of drug.

d Half-life (min) on exposure to human liver microsomes; studies conducted by BioDuro LLC.

e Oral bioavailability (%) in rats; studies conducted by BioDuro LLC.

f See Table 1.

Compounds **3**–**10** explore the use of dimethylthiophene B-ring units. While these are slightly more lipophilic than benzene, the set of compounds prepared had clogP values lower than bedaquiline (between 6.33 and 4.24) and MABA MICs between 0.05 and 0.58 µg/mL. The exception was the much less lipophilic CN derivative **7** (clog P 4.24), which was less active. The 2,3-dimethylthiophene **9**, bearing a more lipophilic 3-F C-unit substituent, had comparable activity to **1** and its cyano counterpart **10**, with a much lower clog P (4.67) was equally potent. The 2-Me and 2-OMe thiophenes **12** and **13** were also effective inhibitors, despite clog P values of around 5.

Given that good activity had been reported for furan **2** (albeit in *M. smegmatis*),^11^ we evaluated a series of both 2- and 3-furyl analogues bearing different C-unit substituents. For the whole dataset (49 compounds, including **1**), despite a wide variation in structure and some MICs being determined using a slightly different protocol (see Table 1), there was a modest positive correlation between potency and lipophilicity (Eq. (1)), as was shown previously for both bedaquiline derivatives^10^ and other classes of *M.tb* inhibitors.^18^

(1) $$\begin{array}{cc} & \log\text{MIC}=-0.53\text{c}\log\text{P}+1.96\\ & \text{n}=46\text{,}\;\text{R}=0.48\text{,}\;\text{p}=0.04\text{,}\;\text{F}=5.1 \end{array}$$

The 2-furyl (**15**–**21**) and 3-furyl (**22**–**28**) compounds had a relatively narrow range of potencies (MICs 0.68–1.96 µg/mL), despite a wide lipophilicity range (clog Ps from 7.51 to 4.02). For this sub-group of similar compounds, high lipophilicity (clog P) correlated with higher potency (MIC) (Eq. (1)). Compounds **29**–**43** of Table 1 explored a variety of 3-pyridyl B-ring units. The clogP values of these again ranged quite widely (from 6.49 to 3.07) but as a class they were more potent than the other B-unit heterocycle compounds, with the majority having MICs < 0.5 µg/mL in both MABA and LORA assays. Finally, the small cohort of 4-pyridyl analogues (**44**–**50**) appeared even more potent, with MICs < 0.05 µg/mL in both assays yet with clog P values around 5 (significantly better than that of **1**). Thus it is possible to go against the overall trend of a positive correlation between MIC and lipophilicity with compounds such as **47** and **48**, which retain the potency of **1** yet have a clog P lower by 2.25 units. This was shown earlier^10^ by studying compounds with varying 6-substituents in the A-ring unit quinoline, where the polar CN substituent lowered overall clog P by 1.38 log units with only an average twofold increase in MIC. In the present study, the seven Br/CN pairs **6/7**, **9**/**10**, **15**/**16**, **22**/**23**, **30/31**, **33/38** and **45/46**, covering a wide range of B-ring units chemistries, likewise showed only an average 2.2-fold increase in MIC.

Table 2 shows that, for both **1** and selected analogues, IC_50_ values for cytotoxicity in mammalian cells (VERO green monkey kidney epithelial cells) were all >10 µg/mL,^19^ and (in the majority of cases) the IC_50_ values for inhibition of the common CYP3A4 metabolizing enzyme were ≥10 µM. The potency of **1** for inhibition of the hERG calcium channel (IC_50_ 0.37 µM)^20^ is seen as a potential liability (cardiovascular toxicity) and changes that attenuated this effect would be beneficial. However, for the compounds tested there was only modest (at best 5-fold; compound **43**) and unpredictable improvement in this parameter. Compound **1** has quite low clearance in human liver microsomes, as reflected in its Cl_int_ and t1/2 values in Table 2, with all of the other analogues having significantly higher rates of clearance. Finally, compound **49** showed an oral bioavailability comparable to that of **1**, despite its significantly lower lipophilicity. Indeed, all of the compounds in Table 2 had acceptable bioavailability.

## Conclusions

This work, part of a study^10^ exploring analogues of **1** with an altered profile of biological properties, has focused on the effects of replacing the phenyl B-ring unit of bedaquiline with heterocycles of differing lipophilicity, in compounds with a smaller C-ring unit; thiophene-, furan- and pyridyl-based B-ring units were studied. In addition to the expected broad positive correlation between lipophilicity and anti-TB potency (Eq. (1)), the 3-pyridyl and especially the 4-pyridyl compounds did seem to have an additional measure of potency, suggesting that further exploration of the B-unit region might be fruitful.

## A Supplementary data

### MOL files

The following ZIP file contains the MOL files of the most important compounds referred to in this article.

MOL files ZIP file containing the MOL files of the most important compounds in this article.
