# Supplementary material for: Synthesis and evaluation of analogues of the tuberculosis drug bedaquiline containing heterocyclic B-ring units
Source: Bioorg Med Chem Lett. 2017 Dec 1;27(23):5190–6. doi: 10.1016/j.bmcl.2017.10.042 (PMC5696560; doi:10.1016/j.bmcl.2017.10.042)
Supplement: Supplementary data 1 [file mmc1.docx]

**Supplementary Material**

Synthesis and evaluation of analogues of the tuberculosis drug bedaquiline containing heterocyclic B-ring units

Peter J. Choi, Hamish S. Sutherland, Amy S.T. Tong, Adrian Blaser, Scott G. Franzblau, Christopher B. Cooper, Manisha U. Lotlikar, Anna M. Upton, Jerome Guillemont, Magali Motte, Laurence Queguiner, Koen Andries, Walter Van den Broeck, William A. Denny, Brian D. Palmer

Contents

| **Heading** | **page** |
| --- | --- |
| New compounds of Table 1 | 1 |
| New Compounds of Scheme 2 | 3 |
| New Compounds of Scheme 3 | 4 |
| New Compounds of Scheme 4 | 5 |
| New Compounds of Scheme 5 | 7 |
| 6-Bromo compounds of Table 1 in Schemes 1-4 | 8 |
| 6-Cyano compounds of Table 1 in Scheme 6 | 9 |

**New Compounds of Scheme 1**

**6-Bromo-3-((2,5-dimethylthiophen-3-yl)methyl)-2-methoxyquinoline (54)**.

A solution of 2,2,6,6-tetramethylpiperidine (1.89 mL, 11.1 mmol) in dry THF (12 mL) was cooled to -40 °C, *n*-BuLi (4.45 mL of a 2.5N solution in hexane, 11.1 mmol) was added and the solution was stirred at -40 °C for 15 min, then cooled to -78 °C. A solution of 6-bromo-2-methoxyquinoline (**51**) (2.20 g, 9.28 mmol) in THF (10 mL) was added dropwise, the orange solution was stirred at -78 °C for 1.5 h, then a solution of 2,5-dimethylthiophene-3-carbaldehyde (**52)** (1.30 g, 9.27 mmol) in THF (10 mL) was added. The mixture was stirred at -78 °C for 3 h, then acetic acid (1.60 mL, 28.0 mmol) was added and the solution was allowed to warm to r.t.. The mixture was partitioned between EtOAc and water, and the organic fraction was dried and evaporated. Chromatography with DCM:hexanes (1:3) eluted starting materials, then elution with DCM:hexanes (1:1) gave 6-bromo-2-methoxyquinolin-3-yl)(2,5-dimethylthiophen-3-yl)methanol (**53**) as a white solid (1.97 g, 56%). M.p. 119-122 °C. ^1^H NMR (CDCl_3_) δ 7.93 (s, 1H), 7.88 (d, *J* = 2.1 Hz, 1H), 7.70 (d, *J* = 8.4 Hz, 1H), 7.65 (dd, *J* = 8.4, 2.1 Hz, 1H), 6.51 (d, *J* = 1.0 Hz, 1H), 6.04 (dd, *J* = 3.3, 0.8 Hz, 1H), 4.07 (s, 3H), 2.67 (d, *J* = 3.4 Hz, 1H), 2.43 (s, 3H), 2.34 (s, 3H). Found: [M+H]=378.2.

Triethylsilane (6.8 mL, 42.1 mmol) was added to a solution of **53** (1.99 g, 5.26 mmol) and TFA (3.9 mL, 52.5 mmol) in DCM (50 mL) at 0 °C, and the solution was stirred for 0.5 h at 0 °C then at r.t. for 2 h. The solution was cooled to 0 °C, quenched with sat. aq. NaHCO_3_ and partitioned between DCM and water. Column chromatography (1:3 DCM:hexanes) gave 6-bromo-3-((2,5-dimethylthiophen-3-yl)methyl)-2-methoxyquinoline (**54**) as a white solid (1.70 g, 89%). M.p. 120-121 °C. ^1^H NMR (CDCl_3_) δ 7.76 (d, *J* = 2.2 Hz, 1H), 7.69 (d, *J* = 8.9 Hz, 1H), 7.61 (dd, *J* = 8.9, 2.2 Hz, 1H), 7.40 br s, 1H), 6.42 (s, 1H), 4.10 (s, 3H), 3.83 (s, 2H), 2.39 (s, 3H), 2.31 (s, 3H). Found: [M+H]=362.2.

Similar reaction of **51** with 2,3-dimethylthiophene-3-carbaldehyde (**56**) gave 6-bromo-2-methoxyquinolin-3-yl)(2,3-dimethylthiophen-3-yl)methanol (**57**) (58%). M.p. 151-153 °C. 1H NMR (CDCl3) δ 8.06 (s, 1H), 7.89 (d, *J* = 2.0 Hz, 1H), 7.71 (d, *J* = 8.9 Hz, 1H), 7.67 (dd, *J* = 8.9, 2.1 Hz, 1H), 6.59 (s, 1H), 6.14 (d, *J* = 5.3 Hz, 1H), 4.07 (s, 3H), 2.96 (d, *J* = 5.3 Hz, 1H), 2.29 (s, 3H), 2.05 (s, 3H). Found: [M+H] = 380.1, 378.0.

This was reduced as above to give 6-bromo-2-methoxyquinolin-3-yl)(2,3-dimethylthiophen-3-yl)methanol (**58**) (31%). M.p. 105-107 °C. ^1^H NMR (CDCl_3_) δ 7.79 (d, *J* = 2.2 Hz, 1H), 7.69 (d, *J* = 8.9 Hz, 1H), 7.63 (s, 1H), 7.61 (dd, *J* = 8.9, 2.2 Hz, 1H), 6.53 (s, 1H), 4.10 (s, 3H), 4.09 (s, 2H), 2.29 (s, 3H), 2.08 (s, 3H). Found: [M+H] = 364.1, 362.1.

Similar reaction of **51** with 5-methylthiophene-2-carbaldehyde (**59**) gave 6-bromo-2-methoxyquinolin-3-yl)(5-methylthiophen-2-yl)methanol (**60**) (55%). M.p. 99-101 °C; ^1^H NMR (CDCl_3_) δ 8.00 (s, 1H), 7.88 (d, *J* = 2.1 Hz, 1H), 7.71 (d, *J* = 8.8 Hz, 1H), 7.67 (dd, *J* = 8.9, 2.2 Hz, 1H), 6.71 (d, *J* = 3.4 Hz, 1H), 6.59 (dd, *J* = 3.4, 1.0 Hz, 1H), 6.18 (d, *J* = 5.3 Hz, 1H), 4.07 (s, 3H), 3.01 (d, *J* = 5.4 Hz, 1H), 2.44 (d, *J* = 1.0 Hz, 3H). Found: [M+H] = 366.1, 364.0.

This was reduced as above to give 6-bromo-2-methoxy-3-((5-methylthiophen-2-yl)methyl)quinoline (**61**) (79%). M.p. 81-82 °C. ^1^H NMR (CDCl_3_) δ 7.78 (d, *J* = 2.2 Hz, 1H), 7.69 (d, *J* = 8.8 Hz, 1H), 7.62 (br s, 1H), 7.61 (dd, *J* = 8.8, 2.2 Hz, 1H), 6.65 (d, *J* = 3.3 Hz, 1H), 6.59 (d, *J* = 3.3 Hz, 1H), 4.13 (br s, 2H), 4.10 (s, 3H), 2.44 (s, 3H). Found: [M+H]=348.2.

Similar reaction of **51** with 5-methoxythiophene-2-carbaldehyde (**62**) gave (6-bromo-2-methoxyquinolin-3-yl)(5-methoxythiophen-2-yl)methanol (**63**) (53%). M.p. 211-214 °C. ^1^H NMR (CDCl_3_) δ 8.01 (s, 1H), 7.89 (d, *J* = 2.0 Hz, 1H), 7.71 (d, J =8.9 Hz, 1H), 7.67 (dd, *J* = 8.9, 2.1 Hz, 1H), 6.53 (dd, *J* = 3.8, 0.8 Hz, 1H), 6.09 (d, *J* = 5.2 Hz, 1H), 6.02 (d, *J* = 3.8 Hz, 1H), 4.07 (s, 3H), 3.84 (s, 3H), 3.00 (d, *J* = 5.2 Hz, 1H). Found: [M - OMe] = 350.0, 348.0.

This was reduced as above to give 6-bromo-2-methoxy-3-((5-methoxythiophen-2-yl)methyl)quinolone (**64**) (69%). M.p. 61-63 °C. ^1^H NMR (CDCl_3_) δ 7.79 (d, *J* = 2.2 Hz, 1H), 7.69 (d, *J* = 8.9 Hz, 1H), 7.64 (s, 1H), 7.62 (dd, *J* = 8.9, 2.2 Hz, 1H), 6.48 (dt, *J* = 3.8, 1.0 Hz, 1H), 6.03 (d, *J* = 3.8 Hz, 1H), 4.10 (s, 3H), 4.04 (s, 2H), 3.85 (s, 3H). Found: [M+H] = 366.0, 364.1.

**New Compounds of Scheme 2**

**(6-Bromo-2-methoxyquinolin-3-yl)(furan-2-yl)methanol (66)**: A solution of 2,2,6,6-tetramethylpiperidine (5.10 ml, 30.2 mmol) in dry THF (20 ml) was cooled to -30 ^o^C under an atmosphere of dry nitrogen gas. N-Butyllithium (15.1 ml of a 2N solution in cyclohexane, 30.2 mmol) was added dropwise and the solution was stirred at this temperature for 10 min. The solution was cooled to -70 ^o^C and a solution of 6-bromo-2-methoxyquinoline (**51**) (4.00 g, 16.8 mmol) in dry THF (15 ml) was added dropwise. The solution was stirred at -70 to -75 ^o^C for 2 h, then a solution of 2-furaldehyde (**65**) (1.77 ml, 18.4 mmol) in dry THF (10 ml) was added dropwise. After stirring at this temperature for 1 h the cooling bath was removed and the solution was allowed to warm to approximately 0 ^o^C. Water was added and the mixture was extracted into ethyl acetate. The extract was washed with water, dried over sodium sulfate and the solvent was removed under reduced pressure, leaving a yellow oil. The oil was chromatographed on silica. 5% Ethyl acetate/petroleum ether eluted unreacted bromoquinoline (**51**). 25% Ethyl acetate/petroleum ether eluted the product (**66**) as a golden oil (3.68 g (65%). ^1^H NMR (CDCl_3_) δ 8.01 (br s, 1H), 7.88 (d, *J* = 2.1 Hz, 1H), 7.72 (d, *J* = 8.9 Hz, 1H), 7.67 (dd, *J* = 8.9, 2.1 Hz, 1H), 7.40 (m, 1H), 6.34 (dd, *J* = 3.2, 1.8 Hz, 1H), 6.16 (d, *J* = 3.2 Hz, 1H), 6.09 (br s, 1H), 4.06 (s, 3H). Found: [M+H]=335.

**6-Bromo-3-(furan-2-ylmethyl)-2-methoxyquinoline (67)**:To a stirred solution of **66** (1.82 g, 5.45 mmol) in dichloromethane (50 ml) at room temperature was added TFA (4.06 ml, 54.4 mmol), followed by triethylsilane (7.03 ml, 43.0 mmol) and the solution was stirred at room temperature for 1 h. Ice-water was added and the mixture was diluted with more dichloromethane. The organic solution was washed with water, dried over sodium sulfate, and concentrated to dryness under reduced pressure. The residue was adsorbed directly onto silica and chromatographed on silica. Elution with 0-2% ethyl acetate/petroleum ether gave the product (**67**) as a white solid (1.08 g, 62%), which crystallized from methanol as white needles, mp 181-183 ^o^C. ^1^H NMR (CDCl_3_) δ 7.77 (d, *J* = 2.2 Hz, 1H), 7.68 (d, *J* = 8.8 Hz, 1H), 7.61 (dd, *J* = 8.8, 2.2 Hz, 1H), 7.55 (br s, 1H), 7.36 (dd, *J* = 1.9, 0.8 Hz, 1H), 6.34 (dd, *J* = 3.2, 1.9 Hz, 1H), 6.11 (dd, *J* = 3.2, 0.8 Hz, 1H), 4.08 (s, 3H), 4.04 (s, 2H). Found; [M+H]=319.

**6-Bromo-3-(furan-3-ylmethyl)-2-methoxyquinoline (70)**: A solution of 2,2,6,6-tetramethylpiperidine (14.4 mL, 84.8 mmol) in THF (100 mL, dist. Na) at -78°C was treated with *n*-BuLi (33 mL, 2.5 M in hexanes, 82.5 mmol), the solution was then warmed to -20°C for 20 min and then cooled to -78°C. A solution of 6-bromo-2-methoxyquinoline (**51**) (10.0 g, 42.0 mmol) and triisopropylborate (20.0 mL, 87.2 mmol) in THF (100 mL, dist. Na) was added dropwise and the orange solution was stirred for 3 h at -78°C, warmed to -40°C and then quenched with sat. aq. NH_4_Cl (500 mL). The mixture was diluted with water (1 L) and the white precipitate was filtered, triturated with hexanes and dried to give 6-bromo-2-methoxyquinolin-3-yl)boronic acid (**68**) (11.17 g, 94%) as a white solid. ^1^H NMR (DMSO-d_6_) δ 8.44 (s, 1H), 8.15-8.18 (m, 3H), 7.76 (dd, J = 8.8, 2.3 Hz, 1H), 7.68 (d, J = 8.9 Hz, 1H), 3.99 (s, 3H). Found: [M-OH+OMe]=296.2.

A mixture of (6-bromo-2-methoxyquinolin-3-yl)boronic acid (**68**), (1.50 g, 7.45 mmol), freshly prepared 3-(bromomethyl)furan (**69**) (1.80 g, 11.07 mmol) and Cs_2_CO_3_ (4.85 g, 14.90 mmol) in toluene (20 ml) and DMF (10 ml) was degassed by bubbling dry nitrogen gas through it for 15 min. Tetrakis(triphenylphosphine)palladium (0.30 g, 0.27 mmol) was then added and the mixture was stirred under an atmosphere of nitrogen at 90 ^o^C for 5 h. The mixture was then cooled, diluted with ethyl acetate and washed with water. The organic portion was dried over sodium sulfate and the solvent was removed under reduced pressure. The residue was chromatographed on silica. Elution with 2% ethyl acetate/petroleum ether gave the product (**70**), which crystallised from methanol as a white solid (0.74 g, 31%), mp 78 ^o^C. ^1^H NMR (CDCl_3_) δ 7.77 (d, *J* = 2.2 Hz, 1H), 7.69 (d, *J* = 8.9 Hz, 1H), 7.61 (dd, *J* = 8.9, 2.2 Hz, 1H), 7.58 (br s, 1H), 7.40 (t, *J* = 1.7 Hz, 1H), 7.31 (m, 1H), 6.30 (m, 1H), 4.09 (s, 3H), 3.83 (br s, 2H). Found: [M+H]=319. Found C: 55.89, H: 3.74: N, 4.37. C_15_H_12_BrNO_2_.0.25H_2_O requires C: 55.83, H: 3.90, N: 4.34%.

**New Compounds of Scheme 3**

**6-Bromo-2-methoxy-3-((2-methoxypyridin-3-yl)methyl)quinoline (72)**

A mixture of **68** (1.00 g, 3.55 mmol), 3-(chloromethyl)-2-methoxypyridine (**71**) (0.68 g, 4.31 mmol) and Cs_2_CO_3_ (2.31 g, 7.09 mmol) in toluene:DMF (60 mL, 2:1) was degassed under N_2_, then Pd(PPh_3_)_4_ (0.082 g, 0.071 mmol) was added, and the mixture heated at 80 °C for 4 h. The reaction mixture was cooled to r.t., filtered through a plug of Celite, water (150 mL) was added and extracted with EtOAc (3 x 100 mL). The combined organic layers were washed with brine (100 mL), dried over Na_2_SO_4_, filtered and concentrated under reduced pressure to obtain a yellow residue. Purification by flash column chromatography using hexanes:EtOAc (9:1) gave 6-bromo-2-methoxy-3-((2-methoxypyridin-3-yl)methyl)quinoline (**72** ) as a white solid (0.94 g, 74%). ^1^H NMR (CDCl_3_) δ 8.08 (dd, J = 5.0, 1.9 Hz, 1H), 7.77 (d, J = 2.2 Hz, 1H), 7.68 (d, J = 8.9 Hz, 1H), 7.61 (dd, J = 8.9, 2.2 Hz, 1H), 7.53 (s, 1H), 7.38 (dd, J = 7.2, 1.9 Hz, 1H), 6.83 (dd, J = 7.2, 5.0 Hz, 1H), 4.07 (s, 3H), 3.96 (s, 2H), 3.95 (s, 3H). Found: [M+H]=359.6.

**3-((6-Bromo-2-methoxyquinolin-3-yl)methyl)-*N,N*-dimethylpyridin-2-amine (75)**

Similar reaction of **51** with 2-(dimethylamino)nicotinaldehyde (**73**) gave (6-bromo-2-methoxyquinolin-3-yl)(2-(dimethylamino)pyridin-3-yl)methanol (**74**) (56%): ^1^H NMR (CDCl_3_) δ 8.31 (dd, J = 4.8, 1.9 Hz, 1H), 8.05 (s, 1H), 7.88 (d, J = 2.1 Hz, 1H), 7.73 (d, J = 8.9 Hz, 1H), 7.68 (dd, J = 8.9, 2.2 Hz, 1H), 7.31 (dd, J = 7.5, 1.8 Hz, 1H), 6.95 (dd, J = 7.7, 4.8 Hz, 1H), 6.31 (s, 1H), 6.07 (s, 1H), 4.06 (s, 3H), 2.82 (s, 6H). Found: [M+H]= 388.7.

Compound **74** was then reduced as above to give 3-((6-bromo-2-methoxyquinolin-3-yl)methyl)-*N,N*-dimethylpyridin-2-amine (**75**) (76%): ^1^H NMR (CDCl_3_) δ 8.22 (dd, J = 4.8, 1.8 Hz, 1H), 7.75 (d, J = 2.2 Hz, 1H), 7.71 (d, J = 8.9 Hz, 1H), 7.64 (dd, J = 8.9, 2.2 Hz, 1H), 7.50 (s, 1H), 7.23 (dd, J = 7.4, 1.9 Hz, 1H), 6.83 (dd, J = 7.4, 4.8 Hz, 1H), 4.07 (s, 3H), 4.06 (s, 2H), 2.83 (s, 6H). Found: [M+H]= 372.7.

**5-((6-bromo-2-methoxyquinolin-3-yl)methyl)-*N,N*-dimethylpyridin-2-amine (80)**

Similar reaction of **51** with 6-(dimethylamino)nicotinaldehyde (**76**) gave (6-bromo-2-methoxyquinolin-3-yl)(6-(dimethylamino)pyridin-3-yl)methanol (**78**) (79%): ^1^H NMR (CDCl_3_) δ 8.18 (d, J = 2.4 Hz, 1H), 7.99 (s, 1H), 7.87 (d, J = 2.0 Hz, 1H), 7.69 (d, J = 8.9 Hz, 1H), 7.65 (dd, J = 8.9, 2.1 Hz, 1H), 7.43 (dd, J = 8.8, 2.5 Hz, 1H), 6.48 (d, J = 8.7 Hz, 1H), 5.96 (d, J = 3.4 Hz, 1H), 4.03 (s, 1H), 3.08 (s, 6H), 2.72 (d, J = 4.0 Hz, 1H).

Compound **78** was reduced as above to give 5-((6-bromo-2-methoxyquinolin-3-yl)methyl)-*N,N*-dimethylpyridin-2-amine (**80**) (70%): ^1^H NMR (CDCl_3_) δ 8.10 (d, J = 2.0 Hz, 1H), 7.74 (d, J = 2.2 Hz, 1H), 7.68 (d, J = 8.8 Hz, 1H), 7.61 (dd, J = 8.9, 2.2 Hz, 1H), 7.49 (s, 1H), 7.31 (dd, J = 8.7, 2.4 Hz, 1H), 6.49 (d, J = 8.7 Hz, 1H), 4.08 (s, 3H), 3.87 (s, 2H), 3.08 (s, 6H). Found: [M+H]= 372.7.

**5-((6-Bromo-2-methoxyquinolin-3-yl)methyl)-*N,N*-diethylpyridin-2-amine (81)**

Similar reaction of **51** with 6-(diethylamino)nicotinaldehyde (**77**) gave (6-bromo-2-methoxyquinolin-3-yl)(6-(diethylamino)pyridin-3-yl)methanol (**79**) (57%): ^1^H NMR (CDCl_3_) δ 8.15 (d, J = 2.4 Hz, 1H), 8.01 (s, 1H), 7.88 (d, J = 2.1 Hz, 1H), 7.69 (d, J = 8.8 Hz, 1H), 7.65 (dd, J = 8.9, 2.1 Hz, 1H), 7.40 (dd, J = 8.9, 2.5 Hz, 1H), 6.42 (d, J = 8.9 Hz, 1H), 5.94 (d, J = 3.7 Hz, 1H), 4.04 (s, 3H), 3.53-3.47 (m, 4H), 2.65 (d, J = 3.9 Hz, 1H), 1.17 (t, J = 7.0 Hz, 6H).

Compound **79** was reduced as above to give 5-((6-bromo-2-methoxyquinolin-3-yl)methyl)-*N,N*-diethylpyridin-2-amine (**81**) (76%): ^1^H NMR (CDCl_3_) δ 8.07 (d, J = 2.1 Hz, 1H), 7.76 (d, J = 2.2 Hz, 1H), 7.67 (d, J = 8.8 Hz, 1H), 7.61 (dd, J = 8.8, 2.2 Hz, 1H), 7.52 (s, 1H), 7.27 (dd, J = 8.7, 2.4 Hz, 1H), 6.43 (dd, J = 8.7, 0.3 Hz, 1H), 4.09 (s, 3H), 3.85 (s, 2H), 3.50 (q, J = 7.0 Hz, 4H), 1.18 (t, J = 7.0, 6H). Found: [M+H]= 400.8.

**6-Bromo-2-methoxy-3-((6-(methylthio)pyridin-3-yl)methyl)quinoline (84)**

Similar reaction of **68** with 5-(chloromethyl)-2-(methylthio)pyridine (**82**) gave 6-bromo-2-methoxy-3-((6-(methylthio)pyridin-3-yl)methyl)quinoline (**84**) (59%): ^1^H NMR (CDCl_3_) δ 8.37 (d, J = 1.8 Hz, 1H), 7.77 (d, J = 2.1 Hz, 1H), 7.69 (d, J = 8.9 Hz, 1H), 7.63 (dd, J = 8.9, 2.2 Hz, 1H), 7.55 (s, 1H), 7.35 (dd, J = 8.3, 2.3 Hz, 1H), 7.13 (dd, J = 8.2, 0.6 Hz, 1H), 4.07 (s, 3H), 3.96 (s, 2H), 2.56 (s, 3H). Found: [M+H]= 375.1.

**6-Bromo-3-((6-(ethylthio)pyridin-3-yl)methyl)-2-methoxyquinoline (85)**

Similar reaction of **68** with 5-(chloromethyl)-2-(ethylthio)pyridine (**83**) gave 6-bromo-3-((6-(ethylthio)pyridin-3-yl)methyl)-2-methoxyquinoline (**85**) (62%): ^1^H NMR (CDCl_3_) δ 8.36 (d, J = 1.7 Hz, 1H), 7.77 (d, J = 2.1 Hz, 1H), 7.69 (d, J = 8.8 Hz, 1H), 7.63 (dd, J = 8.8, 2.2 Hz, 1H), 7.55 (s, 1H), 7.34 (dd, J = 8.2, 2.3 Hz, 1H), 7.11 (dd, J = 8.3, 0.6 Hz, 1H), 4.07 (s, 3H), 3.95 (s, 2H), 3.16 (q, J = 7.3 Hz, 2H), 1.37 (t, J = 7.3 Hz, 3H). Found: [M+H]= 389.6.

**New Compounds of Scheme 4**

**(5-Bromomethyl)-2,3-dimethoxypyridine (88)**

*n*-BuLi (4.90 mL, 2.0 M in cyclohexanes, 35.2 mmol) was added to a solution of 5-bromo-2,3-dimethoxypyridine (7.00 g, 32.1 mmol) in dry THF (100 mL) at -35 °C, the cream coloured precipitate was stirred at -35 °C for 0.5 h then DMF (4.90 mL, 63.7 mmol) was added. The mixture was stirred at -35 °C for 1 h, then quenched with water and partitioned between EtOAc and water, the organic fraction was dried and evaporated. The crude aldehyde was dissolved in MeOH (50 mL) and cooled to -40 °C, NaBH_4_ (1.20 g, 32 mmol) was added and the mixture was stirred at -40 °C for 1 h and then quenched with water. The mixture was partitioned between EtOAc and water, the organic fractions were dried and evaporated. Column chromatography using hexanes:EtOAc (2:1) gave (5,6-dimethoxypyridin-3-yl)methanol (4.02 g, 74%). ^1^H NMR (CDCl_3_) δ 7.67 (d, *J* = 1.9 Hz, 1H), 7.13 (d, *J* = 1.9 Hz, 1H), 4.63 (d, *J* = 5.4 Hz, 2H), 4.02 (s, 3H), 3.90 (s, 3H), 1.77 (t, *J* = 5.4 Hz, 1H). Found: [M+H] = 170.2.

A solution of (5,6-dimethoxypyridin-3-yl)methanol (3.98 g, 23.6 mmol) in DCM (anhydrous, 80 mL) at 0 °C was treated with triethylamine (6.6 mL, 47.4 mmol) and then mesyl chloride (2.73 mL, 35.3 mmol), the mixture was stirred at 0 °C for 1 h and then partitioned between DCM and water. The organic fraction was dried and evaporated and the residue was dissolved in acetone (100 mL), LiBr (10.2 g, 117 mmol) was added and the mixture was refluxed for 1 h and then evaporated. The residue was partitioned between DCM and water; the organic fraction was dried and evaporated. Column chromatography (DCM) gave (5-bromomethyl)-2,3-dimethoxypyridine (**88**) (3.36 g, 61%) which was contaminated with the corresponding chloro derivative (0.30 g, 7%). ^1^H NMR (CDCl_3_) δ 7.73 (d, *J* = 2.0 Hz, 1H), 7.07 (d, *J* = 2.0 Hz, 1H), 4.47 (s, 2H), 4.02 (s, 3H), 3.90 (s, 3H). Found: [M+H] = 232.4

**6-Bromo-3-((5,6-dimethoxypyridin-3-yl)methyl)-2-methoxyquinoline (89)**

A mixture of **68** (4.02 g, 14.3 mmmol), **88** (3.34 g, 14.4 mmol) and Cs_2_CO_3_ (9.3 g, 28.5 mmol) in toluene (80 mL, anhydrous) and DMF (40 mL, anhydrous) was purged with nitrogen. Pd(PPh_3_)_4_ (0.33 g, 0.3 mmol) was added, the mixture was purged with nitrogen, then heated to 80 °C under nitrogen for 4 h. The reaction was partitioned between EtOAc and water and the organic fraction was dried and evaporated. Column chromatography (95:5 DCM:EtOAc) gave the product which was recrystallized from MeOH to give **89** (3.85 g, 69%). M.p. 131-133 °C. ^1^H NMR (CDCl_3_) δ 7.77 (d, *J* = 2.2 Hz, 1H), 7.70 (d, *J* = 8.9 Hz, 1H), 7.65 (d, *J* = 1.9 Hz, 1H), 7.63 (dd, *J* = 8.9, 2.2 Hz, 1H), 7.52 (s, 1H), 6.92 (d, *J* = 1.9 Hz, 1H), 4.09 (s, 3H), 4.02 (s, 3H), 3.95 (s, 2H), 3.83 (s, 3H). Found: [M+H] = 389.1

**4-(Bromomethyl)-2,3-dimethoxypyridine (90)**

A warm solution of 2,3-dimethoxyisonicotinaldehyde (0.68 g, 4.10 mmol) in EtOH (5 mL) was added dropwise to a suspension of NaBH_4_ (0.08 g, 2.04 mmol) in EtOH (10 mL) at -40 °C, gas evolution occurred and the mixture was stirred at -40 °C for 45 min The mixture was quenched with brine (10 mL) and diluted with water (10 mL), then extracted with ether (3 x 50 mL). Column chromatography (1:1 hexanes:EtOAc) gave (2,3-dimethoxypyridin-4-yl)methanol (0.69 g, 98%) as a white solid. M.p. 87-90 °C. ^1^H NMR (CDCl_3_) δ 7.89 (d, *J* = 5.1 Hz, 1H), 6.93 (d, *J* = 5.1 Hz, 1H), 4.73 (d, *J* = 6.1 Hz, 2H), 4.01 (s, 3H), 3.88 (s, 3H), 2.15 (t, *J* = 6.1 Hz, 1H). Found: [M+H] = 170.2.

A solution of (2,3-dimethoxypyridin-4-yl)methanol (2.83 g, 16.8 mmol) and triethylamine (4.70 mL, 33.7 mmol) in DCM (50 mL, anhydrous) was cooled to 0°C, then treated with mesyl chloride (1.95 mL, 25.2 mmol). The cloudy suspension was stirred at 0°C for 1 h and partitioned between DCM and water. The organic fraction was dried and evaporated to give the crude mesylate. The crude mesylate was dissolved in acetone (100 mL) and LiBr (14.5 g, 167 mmol) was added. The mixture was refluxed for 1h, evaporated and the residue was partitioned between DCM and water. The organic fraction was dried and evaporated. Column chromatography (DCM) gave **90** (3.53 g, 91%) as a colourless oil. ^1^H NMR (CDCl_3_) δ 7.85 (d, *J* = 5.2 Hz, 1H), 6.87 (d, *J* = 5.2 Hz, 1H), 4.45 (s, 2H), 4.01 (s, 3H), 3.95 (s, 3H). Found: [M+H ]= 232.4

**6-Bromo-3-((2,3-dimethoxypyridin-4-yl)methyl)-2-methoxyquinoline (91)**

A mixture of **68** (10.28 g, 36.5 mmol), **90** (8.64 g, 36.5 mmol) and Cs_2_CO_3_ (24.0 g, 73.7 mmol) in DMF:toluene (1:2, 200 mL) was purged with nitrogen. Pd(PPh_3_)_4_ (0.84 g, 0.73 mmol) was added and the mixture was heated to 80 °C for 3 h under nitrogen. The mixture was partitioned between EtOAc and water and the aqueous layer was extracted with EtOAc. The organic fraction was dried and evaporated, column chromatography using a gradient of 3:1 hexanes:DCM to 95: 5 DCM:EtOAc gave **91** (8.65 g, 61%). ^1^H NMR (CDCl_3_) δ 7.80 (d, *J* = 5.2 Hz, 1H), 7.77 (d, *J* = 2.2 Hz, 1H),7.69 (d, *J* = 8.9 Hz, 1H), 7.62 (dd, *J* = 8.9, 2.2 Hz, 1H), 7.53 (s, 1H), 6.66 (d, *J* = 5.2 Hz, 1H), 4.07 (s, 3H), 4.03 (s, 2H), 4.02 (s, 3H), 3.80 (s, 3H). Found: [M+H] = 389.1

**New compounds of Scheme 5**

**Route A:**

**1-(Benzo[d][1,3]dioxol-4-yl)-3-(dimethylamino)propan-1-one (55i)**

A mixture of 1-(benzo[d][1,3]dioxol-4-yl)ethan-1-one (4.8 g, 29.3 mmol), dimethylamine HCl (3.08 g, 38 mmol) and paraformaldehyde (1.14 g, 38 mmol) in EtOH (100 mL) and HCl (0.8 mL) was refluxed in a sealed tube for 18 h. The solvent was evaporated and the solid residue was partitioned between EtOAc and water. The aqueous portion was basified with 2M NaOH and then extracted with EtOAc (3 x 100 mL). The organic fractions were washed with water and brine, then dried over Na2SO4 and evaporated to give 1-(Benzo[d][1,3]dioxol-4-yl)-3-(dimethylamino)propan-1-one **(55i)** (1.80 g, 28%). ^1^H NMR (CDCl_3_) δ 7.39 (dd, *J* = 8.2, 1.2 Hz, 1H), 6.98 (dd, *J* = 7.6, 1.2 Hz, 1H), 6.88 (t, J = 7.9 Hz, 1H), 6.08 (s, 2H), 3.15 (t, J = 7.4 Hz, 2H), 2.74 (t, J = 7.4 Hz, 2H), 2.28 (s, 6H). Found: [M+H]=222.2.

**3-(Dimethylamino)-1-(3-methoxyphenyl)propan-1-one (55k)**

3-(Dimethylamino)-1-(3-methoxyphenyl)propan-1-one (**55k**) was synthesised in 82% yield from 1-(3-methoxyphenyl)ethan-1-one using Route A. ^1^H NMR (CDCl_3_) δ 7.55 (ddd, *J* = 7.7, 1.4, 1.1 Hz, 1H), 7.50 (dd, *J* = 2.6, 1.6 Hz, 1H), 7.37 (t, *J* = 7.9 Hz, 1H), 7.11 (ddd, *J* = 8.2, 2.6, 0.9 Hz, 1H), 3.86 (s, 3H), 3.14 (t, *J* = 7.3 Hz, 2H), 2.75 (t, *J* = 7.3 Hz, 2H), 2.29 (s, 6H). Found: [M+H] = 208.2.

**1-(3-(Difluoromethoxy)phenyl)-3-(dimethylamino)propane-1-one (55m)** was similarly synthesised in 25% yield from 1-(3-(difluoromethoxy)phenyl)ethan-1-one using Route A. ^1^H NMR (CD_3_SOCD_3_) δ 7.87 (ddd, *J* = 7.7, 1.4, 1.0 Hz, 1H), 7.70 (bt, *J* = 2.0 Hz, 1H), 7.59 (t, *J* = 8.0 Hz, 1H), 7.45 (dd, *J* = 8.1, 2.4 Hz, 1H), 7.34 (t, *J* = 73.8 Hz, 1H), 3.16 (t, *J* = 7.1 Hz, 2H), 2.59 (t, *J* = 7.1 Hz, 2H), 2.15 (s, 6H). Found: [M+MeOH] = 275.2.

**Route B:**

**3-(Dimethylamino)-1-(6-methoxypyridin-2-yl)propan-1-one (55c)**

A solution of 6-methoxypicolinic acid (2.018g, 13.2 mmol) in DCM (70 mL) was treated with DMF (0.1 mL, 1.3 mmol) and oxalyl chloride (1.34 mL, 15.8 mmol), after 1 h the solution was cooled to 0 °C and *N*,*O*-dimethylhydroxylamine HCl (1.42 g, 14.6 mmol) and pyridine (2.34 mL, 28.9 mmol) were added. The mixture was stirred at room temperature for 18 h, sat. aq. NaHCO_3_ (200 mL) was added and the mixture was partitioned between DCM and water. The organic fractions were dried and evaporated, column chromatography (97.5:2.5 DCM:MeOH) gave *N*,6-dimethoxy-*N*-methylpicolinamide (2.372 g, 92%) as an oil. ^1^H NMR (CD_3_SOCD_3_) δ 7.86 (dd, *J* = 8.3, 7.3 Hz, 1H), 7.66 (dd, *J* = 7.3, 0.7 Hz, 1H), 7.05 (dd, *J* = 8.3, 0.7 Hz, 1H), 3.90 (s, 3H), 3.28 (bs, 3H). Found: [M+H] = 197.2.

Vinyl magnesium bromide (1M in THF, 35 mL, 35 mmol) was added to a solution of *N*,6-dimethoxy-*N*-methylpicolinamide (2.321 g, 11.8 mmol) in anhydrous THF (120 mL) at 0 °C, the solution was stirred at room temperature for 1 h, dimethylamine (2M in THF, 35 mL, 70 mmol) and water (30 mL) were added sequentially. The solution was stirred at room temperature for 1 h then extracted with toluene (200 mL). The aqueous fraction was extracted with toluene (2 x 200 mL) and ethyl acetate (100 mL). The organic fractions were combined, dried and evaporated, the residue was dissolved in toluene (200 mL) and filtered through celite, and evaporation gave 3-(dimethylamino)-1-(6-methoxypyridin-2-yl)propan-1-one (**55c**) (1.688 g, 68%) which was used without further purification. ^1^H NMR (CDCl_3_) δ 7.69 (t, *J* = 7.3 Hz, 1H), 7.64 (dd, *J* = 7.3, 1.0 Hz, 1H), 6.93 (dd, *J* = 8.0, 1.0 Hz, 1H), 3.99 (s, 3H), 3.36 (t, *J* = 7.2 Hz, 2H), 2.77 (t, *J* = 7.2 Hz, 2H), 2.29 (s, 3H). Found: [M+H] = 209.2.

**3-(Dimethylamino)-1-(2-methoxypyridin-3-yl)propan-1-one (55e)**

Similarly was prepared starting from 2-methoxynicotinic acid, *N*,2-dimethoxy-*N*-methylnicotinamide in 61% yield. ^1^H NMR (CDCl_3_) δ 8.22 (dd, *J* = 5.0, 1.8 Hz, 1H), 7.60 (bd, *J* = 6.3 Hz, 1H), 6.93 (dd, *J* = 7.2, 5.0 Hz, 1H), 3.99 (s, 3H), 3.54 (bs, 3H), 3.33 (bs, 3H). Found: [M+H]=197.2.

The Weinreb amide was similarly converted to 3-(dimethylamino)-1-(2-methoxypyridin-3-yl)propan-1-one (**55e**) in 95% yield. ^1^H NMR (CDCl_3_) δ 8.30 (dd, *J* = 4.8, 2.0 Hz, 1H), 8.08 (dd, *J* = 7.6, 2.0 Hz, 1H), 6.98 (dd, *J* = 7.5, 4.8 Hz, 1H), 4.05 (s, 3H), 3.21 (t, *J* = 7.1 Hz, 2H), 2.71 (t, *J* = 7.1 Hz, 2H), 2.27 (s, 6H). Found: [M+H]=209.2.

**3-(Dimethylamino)-1-(6-fluoropyridin-2-yl)propan-1-one (55g)**

Similarly was prepared starting from 6-fluoropicolinic acid, 6-fluoro-*N*-methoxy-*N*-methylpicolinamide in 67% yield. ^1^H NMR (CDCl_3_) δ 7.89 (q, *J* = 7.8 Hz, 1H), 7.58 (bs, 1H), 7.03 (ddd, *J* = 8.3, 2.8, 0.5 Hz, 1H), 3.80 (bs, 3H), 3.39 (bs, 3H). Found: [M+H]=185.2.

The Weinreb amide was similarly converted into 3-(dimethylamino)-1-(6-fluoropyridin-2-yl)propan-1-one (**55g**) in 100% yield. ^1^H NMR (CDCl_3_) δ 7.96-7.93 (m, 2H), 7.15-7.11 (m, 1H), 3.32 (t, *J* = 7.1 Hz, 2H), 2.76 (t, *J* = 7.1 Hz, 2H), 2.28 (s, 6H). Found: [M+H]=185.2.

**3-(Dimethylamino)-1-(pyridin-2-yl)propan-1-one (55j)**

Similarly was prepared starting from picolinic acid, *N*-methoxy-*N*-methylpicolinamide in 42% yield. ^1^H NMR (CDCl_3_) δ 8.62 (dd, *J* = 4.7, 1.1 Hz, 1H), 7.79 (td, *J* = 7.7, 1.7 Hz, 1H), 7.67 (bs, 1H), 7.37 (ddd, *J* = 7.6, 4.8, 1.2 Hz, 1H), 3.76 (bs, 3H), 3.41 (bs, 3H). Found: [M+H]=167.2.

The Weinreb amide was similarly converted into 3-(dimethylamino)-1-(pyridin-2-yl)propan-1-one (**55j**) in 97% yield. ^1^H NMR (CDCl_3_) δ 8.68 (ddd, *J* = 4.8, 1.7, 0.9 Hz, 1H), 8.04 (dt, *J* = 7.9, 1.0 Hz, 1H), 7.83 (td, *J* = 7.7, 1.8 Hz, 1H), 7.46 (ddd, *J* = 7.5, 4.8, 1.2 Hz, 1H), 3.40 (t, *J* = 7.2 Hz, 2H), 2.78 (t, *J* = 7.2 Hz, 2H), 2.29 (s, 6H). Found: [M+H]=179.2.

**6-Bromo compounds of Table 1 in Schemes 1-4**

**3-(1-(6-Bromo-2-methoxyquinolin-3-yl)-4-(dimethylamino)-1-(2,5-dimethylthiophen-3-yl)-2-hydroxybutan-2-yl)benzonitrile (3)**

n-BuLi (2.38 mL, 2.5 M in hexanes, 5.95 mmol) was added to a solution of dry diisopropylamine (0.84 mL, 6.0 mmol) in dry THF (12 mL) at -40 °C. The solution was stirred at -40 °C for 15 min. then cooled to -78 °C and a solution of **54** (1.80 g, 4.97 mmol) in dry THF (10 mL) was added. The resultant purple solution was stirred at -78 °C for 1.5 h, then a solution of **55a** (1.005 g, 4.97 mmol) in dry THF (10 mL) was added and the solution was stirred at this temperature for 3 h. Glacial acetic acid (0.85 mL, 14.8 mmol) was added and the mixture was allowed to warm to room temperature. Water was added and the mixture was extracted with ethyl acetate. The extract was washed with water and dried over magnesium sulfate. Removal of solvent under reduced pressure left an oil, which was chromatographed on silica. Elution with DCM gave fore fractions. Elution with 2.5% MeOH/DCM gave impure product, the crude product was then columned with 2.5% MeOH/ethyl acetate to give **3** as a foam, as a 1:1.1 mixture of diastereomers (0.534 g, 19%). ^1^H NMR δ 8.69 (s), 8.44 (s), 7.93 (d, *J* = 2.1 Hz), 7.81 (d, *J* = 2.1 Hz), 7.81-7.74 (bs), 7.68 (d, *J* = 8.9 Hz), 7.73-7.68 (bs), 7.63 (dd, *J* = 8.9, 2.2 Hz), 7.48-7.55 (m), 7.46 (dt, *J* = 7.5, 1.4 Hz), 7.37 (d, *J* = 7.9 Hz), 7.35 (dt, *J* = 7.6, 1.3 Hz), 7.27-7.21 (m), 7.06 (d, *J* = 0.8 Hz), 4.88 (s), 4.83 (s), 4.13 (s), 3.84 (s), 2.49 (s), 2.40 (s), 2.28 (s), 2.26-2.13 (m), 2.10 (s), 2.08 (s), 2.07 (s), 2.02 (s), 2.11-2.10 (m), 1.92 (s), 1.83-1.60 (m). Found: [M+H]=565.3, 567.3.

The mixture of isomers was separated by supercritical fluid (SCF) HPLC at BioDuro LLC (Beijing), to provide the desired *R*,*S* isomer.

The thienyl derivatives (**4**-**6**, **8**, **9**, **11**-**14**) outlined in Scheme 1 were similarly prepared from the appropriate A/B units (**54**, **58**, **61**, **64**) and the C/D units (Mannich bases) **55a**-**55f** of Table 5.

**1-(6-Bromo-2-methoxyquinolin-3-yl)-4-(dimethylamino)-2-(3-fluorophenyl)-1-(furan-3-yl)butan-2-ol (22)**

A solution of dry diisopropylamine (0.65 ml, 4.67 mmol) in dry THF (10 ml) was cooled to -30 oC under an atmosphere of dry nitrogen. N-Butyllithium (2.33 ml of a 2.0 N solution in cyclohexane, 4.67 mmol) was added dropwise, then stirring was continued for a further 15 min. The solution was cooled to -70 to -78 oC and a solution of 70 (1.24 g, 3.89 mmol) in dry THF (6 ml) was added dropwise. The resulting purple solution was stirred at this temperature for 60 min. A solution of 55f (0.76 g, 3.89 mmol) in THF (6 ml) was added dropwise and the mixture was stirred at this temperature for 4 h. Glacial acetic acid (0.70 ml) was added in one portion and the mixture was allowed to warm to room temperature. Water was added and the mixture was extracted with ethyl acetate. The extract was washed with water and dried over sodium sulfate. Removal of the solvent under reduced pressure left an oil, which was chromatographed on silica. Elution with 10% ethyl acetate/petroleum ether gave fore fractions. Elution with 10-20% ethyl acetate/petroleum ether gave the product 22 as a cream-colored foamy solid, as a 1:1 mixture of diastereomers (1.11 g, 55%). 1H NMR (CDCl3) δ 8.45 (s), 8.36 (s), 7.89 (d, J = 2.2 Hz), 7.79 (d, J = 1.8 Hz), 7.71 (d, J = 8.9 Hz), 7.66-7.62 (m), 7.53-7.47 (m), 7.36 (t, J = 1.6 Hz), 7.32-7.24 (m), 7.18 (t, J = 0.8 Hz), 7.15-7.04 (m), 6.91-6.85 (m), 6.76 (d, J = 1.1 Hz), 6.74-6.69 (m), 6.16 (dd, J = 1.7, 0.6 Hz), 4.86 (s), 4.77 (s), 4.16 (s), 3.82 (s), 2.39-2.08 (m), 2.12 (s), 1.97 (s), 1.83-1.60 (m). Found: [M+H]=513, 515.

The mixture of isomers was separated by supercritical fluid (SCF) HPLC at BioDuro LLC (Beijing), to provide the desired *R*,*S* isomer.

The majority of the other compounds of Table 1 were similarly prepared from the appropriate A/B units of Tables 1-4 and the C/D units (Mannich bases) **55a**-**55f** of Table 5.

**6-Cyano compounds of Table 1 in Scheme 6**

**3-(4-(Dimethylamino)-2-(3-fluorophenyl)-1-(furan-3-yl)-2-hydroxybutyl)-2-methoxyquinoline-6-carbonitrile (23)**: To a solution of **22** (0.60 g, 1.17 mmol) in DMF (5 ml) was added powdered zinc (0.03 g, 0.47 mmol), tri-*o-*tolylphosphine (0.03 g, 0.12 mmol) and tris(dibenzylideneacetone)dipalladium (0.11 g, 0.12 mmol). The mixture was degassed by bubbling dry nitrogen through for 10 min, then warmed under an atmosphere of nitrogen to 40 ^o^C for 10 min. Zn(CN)_2_ (0.08 g, 0.65 mmol) was added ant the mixture was warmed to 55 ^o^C and stirred at this temperature for 20 h. After dilution with ethyl acetate the mixture was washed with water, then brine (6 times). The organic extract was dried over sodium sulfate and the solvent was removed under reduced pressure, leaving an oil which was chromatographed on silica. Elution with 50% ethyl acetate/petroleum ether gave the product (**23**) as a foamy, white solid, as a 1:1 mixture of diastereomers (0.52 g, 94%). ^1^H NMR (CDCl_3_) δ 8.54 (s), 8.50 (s), 8.13 (d, *J* = 1.8 Hz), 8.01 (d, *J* = 1.7 Hz), 7.88 (d, *J* = 8.7 Hz), 7.73 (dd, *J* = 8.7, 1.8 Hz), 7.68-7.64 (m), 7.59 (dd, *J* = 8.6, 1.8 Hz), 7.38 (t, *J =* 1.6 Hz), 7.30-7.25 (m, ), 7.22 (br s), 7.16-7.05 (m), 6.91-6.85 (m), 6.77-6.70 (m), 6.21 (d, *J* = 1.1 Hz), 4.85 (s), 4.77 (s), 4.20 (s), 3.86 (s), 2.41-2.28 (m), 2.17 (s), 2.03 (s), 2.01-1.60 (m). Found: [M+H]=460.

The mixture of isomers were separated by supercritical fluid (SCF) HPLC at BioDuro LLC (Beijing), to provide the desired *R*,*S* isomer.

Compounds **10**, **16**, **23**, **31**, **34** and **46** of Table 1 were similarly prepared from the appropriate bromo analogues of Table 1.
